# Measuring three aspects of motivation among health workers at primary level health facilities in rural Tanzania

**DOI:** 10.1371/journal.pone.0176973

**Published:** 2017-05-05

**Authors:** Miho Sato, Deogratias Maufi, Upendo John Mwingira, Melkidezek T. Leshabari, Mayumi Ohnishi, Sumihisa Honda

**Affiliations:** 1 Department of Community-based Rehabilitation Sciences, Nagasaki University Graduate School of Biomedical Sciences, Nagasaki, Japan; 2 School of Tropical Medicine and Global Health, Nagasaki University, Nagasaki, Japan; 3 President’s Office Regional Administration and Local Goverment, Dodoma, Tanzania; 4 Neglected Tropical Diseases Programme, Ministry of Health, Community Development, Gender, Elderly and Children, Dar es Salaam, Tanzania; 5 School of Public Health and Social Sciences, Muhimbili University of Health and Allied Sciences, Dar es Salaam, Tanzania; Chang Gung Memorial Hospital Kaohsiung Branch, TAIWAN

## Abstract

**Background:**

The threshold of 2.3 skilled health workers per 1,000 population, published in the World Health Report in 2006, has galvanized resources and efforts to attain high coverage of skilled birth attendance. With the inception of the Sustainable Development Goals (SDGs), a new threshold of 4.45 doctors, nurses, and midwives per 1,000 population has been identified. This SDG index threshold indicates the minimum density to respond to the needs of health workers to deliver a much broader range of health services, such as management of non-communicable diseases to meet the targets under Goal 3: Ensure healthy lives and promote well-being for all people of all ages. In the United Republic of Tanzania, the density of skilled health workers in 2012 was 0.5 per 1,000 population, which more than doubled from 0.2 per 1,000 in 2002. However, this showed that Tanzania still faced a critical shortage of skilled health workers. While training, deployment, and retention are important, motivation is also necessary for all health workers, particularly those who serve in rural areas. This study measured the motivation of health workers who were posted at government-run rural primary health facilities.

**Objectives:**

We sought to measure three aspects of motivation—Management, Performance, and Individual Aspects—among health workers deployed in rural primary level government health facilities. In addition, we also sought to identify the job-related attributes associated with each of these three aspects. Two regions in Tanzania were selected for our research. In each region, we further selected two districts in which we carried out our investigation. The two regions were Lindi, where we carried out our study in the Nachingwea District and the Ruangwa District, and Mbeya, within which the Mbarali and Rungwe Districts were selected for research. All four districts are considered rural.

**Methods:**

This cross-sectional study was conducted by administering a two-part questionnaire in the Kiswahili language. The first part was administered by a researcher, and contained questions for gaining socio-demographic and occupational information. The second part was a self-administered questionnaire that contained 45 statements used to measure three aspects of motivation among health workers. For analyzing the data, we performed multivariate regression analysis in order to evaluate the simultaneous effects of factors on the outcomes of the motivation scores in the three areas of Management, Performance, and Individual Aspects.

**Results:**

Motivation was associated with marital status (p = 0.009), having a job description (p<0.001), and number of years in the current profession (<1 year: p = 0.043, >7 years: p = 0.042) for Management Aspects; having a job description (p<0.001) for Performance Aspects; and salary scale (p = 0.029) for Individual Aspects.

**Conclusion:**

Having a clear job description motivates health workers. The existing Open Performance Review and Appraisal System, of which job descriptions are the foundation, needs to be institutionalized in order to effectively manage the health workforce in resource-limited settings.

## Introduction

The World Health Report of 2006 entitled “working together for health,” stated that there was an urgent need for the global community to address the crisis in the global health workforce, particularly in sub-Saharan Africa, where the need for health workers is greatest but the shortage is most severe [1]. The same report stated that the minimum density threshold necessary to deliver the most basic health services is 2.3 skilled health workers per 1,000 population [1]. According to the Global Health Workforce Alliance (GHWA), 83 out of 193 countries fall below the threshold of 2.3 skilled health workers per 1,000 population. Forty-six of these countries are in sub-Saharan Africa, including the United Republic of Tanzania, where the density of skilled professionals per 1,000 population is 0.3 [2].

Since the publication of the World Health Report in 2006, the international community, led by the World Health Organization (WHO), has organized three global forums on human resources for health. The first forum was held in Kampala in 2008. The Kampala Declaration and Agenda for Global Action stated a shared vision in which “all people, everywhere have access to a skilled, motivated health worker, within a robust health system.” This vision was shared and restated during the second forum held in Bangkok in 2011 and the third forum in Recife in 2013. The report for the Recife forum presented areas of progress as well as persisting or new challenges [3]. Among the challenges highlighted in the report was keeping health workers motivated in an enabling environment [2].

As a means to address this issue, there has been growing interest in studying the motivation of health workers over the past decade. Health workers in low-income countries often face a challenging work environment, including high patient volume [4], increased workload due to task shifting [5,6], lack of a routine supply of essential medicines [7,8], supervision that is neither routine nor supportive [9–12], and unpaid overtime work [13,14]. These characteristics are more commonly seen among workers in remote areas. While it is understood that a well-motivated health workforce is essential for a functioning health system, efforts to improve the work environment for health workers, particularly in rural areas, have not made as much progress as expected [1,15–17].

Motivation has been defined as “an individual's degree of willingness to exert and maintain an effort towards organizational goals. It is an internal psychological process and a transactional process: worker motivation is the result of the interactions between individuals and their work environment, and the fit between these interactions and the broader societal context [18].” Both qualitative and quantitative evidence suggests that worker performance largely depends on the motivation level [18–20].

Previous literature on the motivation of health workers has demonstrated that both intrinsic and extrinsic factors influence health worker motivation [21–23], whereby intrinsic motivation refers to attributes such as job satisfaction, commitment, intention to leave, sense of burnout, sense of vocation [23], job security, and workload [24]. In contrast, examples of extrinsic factors include salary [25], availability of resources, managerial support, and the policy environment [23]. A systematic review of motivation and retention of health workers in developing countries concluded that financial incentives, career development, and management issues are the core factors affecting health worker motivation [26,27], Similar results have been obtained from studies conducted in sub-Saharan Africa [9,28–34].

Many of the previous studies on health worker motivation took place in hospital settings [21,34–38]. In the present study, the motivation of health workers posted in government-run, first-line primary health facilities (dispensaries and health centers) in four rural and remote districts of mainland Tanzania were measured to identify and understand the factors that influence rural health workers’ motivation.

## Methods

### Study setting

**Fig 1:**
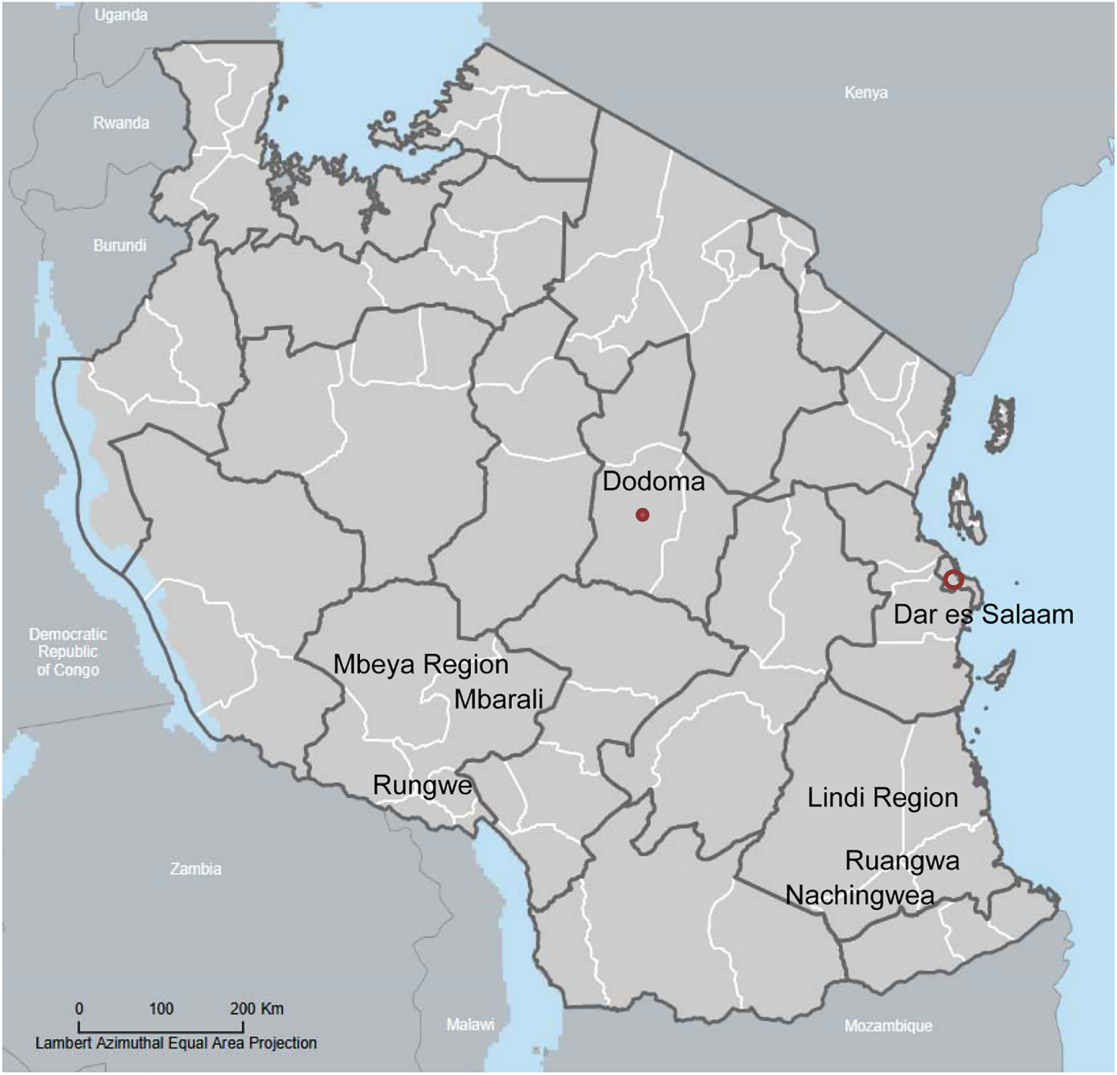
Map of mainland Tanzania showing the location of the four study districts.

The study took place in two districts (Nachingwea and Ruangwa) in the Lindi region in the Southern Zone and two districts (Mbarali and Rungwe) in the Mbeya region in the Southern Highlands of mainland Tanzania (Fig 1).

Among the four study districts, the least populated district was Ruangwa in the Lindi region with 131,080 persons, followed by the Nachingwea district, also in the Lindi region with 178,464 persons. Two districts in the Mbeya region had a population of over 300,000; i.e., the population of the Mbarali district in 2012 was 300,517 and the population of the Rungwe district was 339,157 (Table 1).

**Table 1:** Population, average household size, and number of public health centers/dispensaries in the four study districts.

District	Population (2012)	Average Household size (2012)	The number of public HC+Disp
Nachingwea	178,464	3.7	31
Ruangwa	131,080	3.5	23
Mbarali	300,517	4.3	31
Rungwe	339,157	4.1	60

Using data from the Regional Health Management Team’s annual plan for 2013–2014, the study regions were selected based on the human resources gap in the healthcare sector. A human resources gap refers to the percentage of health worker positions filled relative to the required number of health workers. Based on the average human resources gap (percentage) one district with a gap below average and one district with a gap above average were chosen. For the Mbeya region, whose average human resource gap was 46%, the Rungwe district with a gap of 34% and the Mbarali district with a gap of 47% were selected. Likewise for the Lindi region, whose average human resources gap was 63%, the Nachingwea district with a gap of 56%, and the Ruangwa district with a gap of 73% were selected.

#### Study design

A cross-sectional survey design was used in this study. Data were collected to measure the three aspects of motivation across four districts in Tanzania. These districts included Nachingwea and Ruangwa in the Lindi region, and Mbarali and Rungwe in the Mbeya region. We chose a cross-sectional design to measure the motivation of health workers at the time of administering the questionnaire. Based on the cross-sectional survey results, we then conducted focus group discussions with 70 health workers at 17 public health facilities in the four Districts in order to elucidate the level of motivation from the perspective of the health workers (the results of focus group discussions have been presented elsewhere [39]). The present study is a part of another study to measure the association between teamwork scores among the Council Health Management Teams (CHMTs) and health worker motivation. Therefore, the sample size was calculated to have a statistically significant correlation coefficient between teamwork scores and motivation scores. We used the following values: r = 0.3, alpha (two-sided) = 0.05, beta (one-sided) = 0.2 (See Appendix 1). Hence, the sample size was 85 participants from each of the four districts, including CHMT members and health workers. In total, 329 participants (63 CHMT members and 266 health workers) were recruited. In the present study, we conducted analysis using the data collected from 266 health workers.

#### Participant description

In each district, the study team obtained a list of all health centers and dispensaries from a CHMT. Questionnaires were distributed to a total of 269 health workers who were present at government-run dispensaries and health centers in the study districts. Among them, 263 returned completed questionnaires (response rate, 97.8%). Workers who were on leave or on a business trip did not participate in the study.

### Measures

#### Study instruments

In order to measure the motivation of health workers, the study adopted the Kiswahili version of the Quality of Maternal and Prenatal Care: Bridging the Know-Do Gap (QUALMAT) tool, which was previously applied in three sub-Saharan African countries, including the United Republic of Tanzania [40]. This self-administered questionnaire was composed of two parts. The first part contained questions about the participants’ socio-demographic information and profession. Through personal communication with CHMT members as well as experts in mainland Tanzania, this tool was expanded by adding 11 more items. These additions were necessary to better analyze and understand the motivational factors in this study setting and context.

The second part consisted of 45 statements to measure the motivation of the participants. These statements addressed three aspects of motivation: Management Aspects, Performance Aspects, and Individual Aspects. Management Aspects comprised 16 items that cover five constructs. Performance Aspects comprised 13 items that cover five constructs. Individual Aspects comprised 16 items that cover eight constructs. Motivation scores were aggregated into three Aspects, as in Prytherch *et al*. (2012) [40], which contained a total of 42 statements (14 in Management Aspects, 13 in Performance Aspects, and 15 in Individual Aspects) and was tailored towards health workers who deliver maternal and child health-related services. One statement in Performance Aspects was removed and four new statements were added: two in Management Aspects [10,41–43], one in Performance [10,11,44], and one in Individual Aspects [45]. In addition, two statements in Individual Aspects were modified (see Appendix 2).

The level of motivation was measured on a 4-point Likert scale (1 = strongly disagree, 2 = disagree, 3 = agree, and 4 = strongly agree). The final questionnaire contained 45 items, 12 of which were negative questions. Negative questions were coded in reverse order (i.e. 1 = strongly agree and 4 = strongly disagree). By adding up each score, these scores were aggregated by Aspects (Motivation, Performance, and Individual); higher scores meant higher motivation. Once a draft questionnaire was developed, a pilot test was conducted at two health facilities in a district not located in the study regions, and minor changes were made based on the pilot test. It took approximately 30 to 45 minutes per health worker to complete the questionnaires.

#### Statistical analysis

The reliability of the overall survey instrument was estimated using Cronbach’s alpha (0.826). Pearson’s correlation coefficients were also obtained to measure the correlation among the three Aspects of motivation (Management, Performance, and Individual). Then, multivariate regression analysis was performed to evaluate the simultaneous effects of factors on the outcomes of the motivation scores of the Management, Performance, and Individual Aspects. The most appropriate regression model was selected on the basis of the Akaike Information Criterion. Furthermore, an exploratory factor analysis was conducted to identify latent factors (see Appendix 3).

The Shapiro-Wilk test for normality was used for the normality of data distribution. The Mann-Whitney U test and Kruskal-Wallis test were used to compare the three motivation scores among the groups. All data analyses were carried out using SPSS version 21 (SPSS Inc., Chicago, IL, USA).

### Ethical considerations

All study participants were informed both verbally and in writing of the objectives of the study and were asked to sign a consent form when they agreed to participate in the study. The study was approved by the ethics committees of Nagasaki University Graduate School of Biomedical Sciences (approval number: 12053015), as well as the National Institute of Medical Research of the United Republic of Tanzania (reference number: NIMR/HQ/R.8a/Vol.IX/1446). A research permit was also obtained from the Tanzania Commission for Science and Technology (reference number: 2013-123-NA-2012-124). Confidentiality of data was strictly maintained from the time of data collection throughout the analysis period of the study.

## Results

Table 2 provides the participants’ characteristics. The median age of the participants was 39 years. About half of the participants were single. The median monthly income was 475,000 Tanzanian Shillings (TZS, equivalent to 300USD at the time of data collection). More than a third (34.2%) of the participants were medical attendants (a category of health workers who are at the bottom of the health worker hierarchy in Tanzania, with the lowest level of education) and another third were nurses/midwives (32.7%).

**Table 2:** Characteristics of health workers who participated in the study (N = 263).

Variable	n	%	Median	1st quartile, 3rd quartile
Region	** **	** **	** **	** **
Lindi	121	46.0	** **	** **
Mbeya	142	54.0	** **	** **
District				
Nachingwea (L)	56	21.3		
Ruangwa(L)	65	24.7		
Rungwe(M)	70	26.6		
Mbarali(M)	72	27.4		
Health facility type				
Dispensary	180	68.4		
Health Centre	83	31.6		
Sex				
Male	79	30.0		
Female	184	70.0		
Age group			39	29, 50
20 to 29	65	24.7		
30 to 39	68	25.9		
40 to 49	61	23.2		
≧50	69	26.2		
Marital status				
single	130	49.4		
married	84	31.9		
widowed	18	6.8		
separated	9	3.4		
cohabitation	20	7.6		
Unknown	2	0.8		
Principal provider of financial support to immediate family				
Yes	245	93.2		
No	18	6.8		
Number of dependents			5	4,7
None	14	5.3		
1 to 5	123	46.8		
6 to 10	107	40.7		
11 to 15	17	6.5		
16 and more	2	0.8		
Self-reported take home income (In 10,000 Tsh)			47.5	32.0, 77.2
<199,999 Tsh (TGOHS)	15	5.7		
>200,000 Tsh (TGHS)	246	93.5		
Unknown	2	0.8		
Salary scale				
TGOHS	89	33.8		
TGHS	174	66.2		
Earn extra income				
Yes	31	11.8		
No	231	87.8		
Unknown	1	0.4		
Working in home district				
Yes	70	26.6		
No	192	73.0		
Unknown	1	0.4		
Title				
Health Officer	2	0.8		
Assistant Medical Officer	5	1.9		
Clinical Officer	42	15.8		
Assistant Nursing Officer	10	3.8		
Laboratory Technician	1	0.4		
Clinical Assistant	12	4.5		
Assistant Health Officer	5	1.9		
Laboratory Assistant	4	1.5		
Midwife	4	1.5		
Public Health Nurse	3	1.1		
Nurse Midwife	85	32.7		
Medical Attendant	90	34.2		
Having a job description				
Yes	160	60.8		
No	93	35.4		
Unknown	10	3.8		
Highest level of education before professional training				
Grade 7	86	32.7		
Form 4	158	60.1		
Form 6	17	6.5		
Unknown	2	0.8		
Years of professional training			2.0	1,3
None	58	22.1		
1 year	39	14.8		
2 years	73	27.8		
3 years	42	16		
4 years	36	13.7		
5+ years	15	5.7		
Time in current profession (in years)			6.0	2.1, 13.0
Less than 1 year	20	7.6		
1 to 3 years	75	28.5		
4 to 6 years	54	20.5		
7 to 12 years	47	17.9		
≧13 years	67	25.5		
Time in current health facility (in years)			5.0	2.5, 9.0
Less than 1 year	13	4.9		
1 to 3 years	80	30.4		
4 to 6 years	70	26.6		
7 to 12 years	62	23.6		
≧13 years	38	14.4		
Attended at least one workshop during the past 12 months				
No	95	36.5		
Yes	168	63.5		

Table 3 shows the mean motivation scores by statement for each of the three Aspects. The statement showing more disagreement was “maintenance of broken equipment at this facility is prompt and reliable” (mean: 1.95), whereas the statement demonstrating more agreement was “I try to get on well with the other health staff because it makes the work run more smoothly” (mean: 3.78). There were 25 statements that scored above the overall mean of 3.0, eight of which scored above 3.5 out of 4, which was the highest score.

**Table 3:** Mean scores for the 45-item motivation construct.

Construct			Label			Mean Score (1 to 4)
**I. Management Aspects**						
Work organization	1		This facility provides everything I need to perform well at work.			2.44
	2		There are enough health workers to do the work in this facility			2.07
	3		Too often the referral system does not work efficiently.	**R**		2.64
	4		Maintenance of broken equipment at this facility is prompt and reliable			1.95
Competence strengthening	5		The availability of essential medicines in this facility is poor.	**R**		2.34
	6		Availability of drugs and equipment has improved in the past year			2.52
	7		My job duties and responsibilities are clear and specific			3.30
	8		Relevant policies and guidelines are easy to access at this facility			3.38
	9		I often feel left alone when I have to make difficult decisions about a patient's care.	**R**		2.52
	10		I regularly have access to relevant trainings to keep my skills up to date			2.40
Role of performance	11		My performance is appraised regularly			2.90
	12		Promotions do not depend on how well or badly one works on the job.	**R**		2.50
self-efficacy	13		It is difficult for me to speak openly to my superiors about how things are really going at work.	**R**		2.56
	14		Suggestions made by health workers on how to improve the facility are generally ignored.	**R**		2.41
Provider feels valued/exploited	15		The facility management shows very little concern for me.	**R**		2.82
	16		Our rights as health workers are generally not respected.	**R**		2.20
**II. Performance Aspects**						
Competence strengthening	1		I do not get feedback from my superiors so it is hard to improve my performance.	**R**		2.45
	2		The feedback I get from my co-workers helps me improve my work.			3.33
	3		The feedback I get from CHMT supervisor(s) helps me improve my work.			3.30
Role of performance	4		Good performance is recognised by our superiors.			3.05
	5		This facility has a fair system for rewarding staff.			2.34
	6		Some of the team members work well, yet others do not and so this facility doesn't perform well overall.	**R**		3.18
	7		We do not know how our facility is performing compared to others in the district.	**R**		2.79
	8		Our facility has clear goals that we are working towards.			3.07
	9		I am keen to use any new tools to improve my performance.			3.54
	10		This facility has a good reputation in the community.			3.45
Meaningfulness	11		I understand how my work contributes to the facility's overall goals.			3.44
Attitudes to patients	12		It makes me feel appreciated when patients are grateful.			3.64
Pride/shame	13		I am proud to be working for this health facility			3.07
**III. Individual Aspects**						
Self-efficacy		1	I usually cope well with changes that occur at work.		3.45	
Commitment		2	I intend to leave this facility as soon as I can find another position.	**R**	2.48	
		3	I would recommend to my children that they choose my profession.		3.32	
		4	I am willing to put in a great deal of effort to make this facility successful.		3.72	
General & intrinsic motivation		5	These days I have the morale to work as hard as I can.		3.33	
		6	My profession helps me achieve my goals in life		2.86	
Job satisfaction		7	Overall, I am very satisfied with my work in this facility.		3.24	
		8	I am very satisfied to have a position where one works closely with the community.		3.46	
		9	This job gives me a feeling of achievement and accomplishment.		3.12	
Timeliness and attendance		10	I am punctual about coming to work.		3.56	
		11	I work hard to make sure that no patient has to wait a long time before being seen.		3.55	
Consciousness		12	I am careful not to make errors at work.		3.44	
		14	When I am not sure how to treat a patient's condition I look for information or ask for advice.		3.61	
Competency		13	I have enough training to provide care to patients.		2.90	
Cooperativeness		15	I try to get on well with the other health staff because it makes the work run more smoothly.		3.78	
		16	I get along well with my superiors at work.		3.58	

R indicates the items that are coded in reverse order.

Table 4 demonstrates the difference in aggregated mean motivation scores among variables for each of the three Aspects. Workers who had a job description had significantly higher motivation scores for both Management and Performance Aspects (p<0.001). The duration of working in the current profession also made a difference in motivation scores in Management and Performance Aspects. Those working less than one year and those working 13 years or more had significantly higher motivation scores than those working for 1–12 years (Management Aspects: p<0.027, Performance Aspects: p<0.036). Workers with no dependents had the lowest motivation scores in Management Aspects (p = 0.022).

**Table 4:** Overall motivation mean scores stratified by demographic and professional characteristics.

Variable	I. Management Aspects (N = 238)			II. Performance Aspects (N = 241)			III. Individual Aspects (N = 245)		
	Median	IQR	p-value	Median	IQR	p-value	Median	IQR	p-value
Number of dependents									
None	39	8		42	5		50.5	7	
1 to 5	42	5		41	5		54	6	
6 to 10	40	6		40	5		53	7	
11 to 15	41	6	0.022^b^	40	4	0.915^b^	55.5	7	0.155^b^
Salary scale									
TGOHS	41	7		40.5	5		52	7	
TGHS	41	7	0.581^a^	41	6	0.549^a^	54	7	0.029^a^
Earn extra income									
Yes	43	10		41	6		54	7	
No	41	7	0.067^a^	41	6	0.208^a^	54	7	0.884^a^
Working in home district									
Yes	41	7		41	6		53	7	
No	41	8	0.898^a^	41	7	0.444^a^	54	7	0.441^a^
Qualifications									
4 years of training	43	6		37	8		51	8	
3 years of training	40	6		41	5		55	5	
2 years of training	41	5		41	5		54	5	
1 year of training or none	41	6	0.669^a^	40	4	0.825^a^	52	5	0.014^a^
Having a job description									
Yes	42	7		42	7		53	7	
No	39	6	<0.001^a^	39	6	<0.001^a^	54	7	0.811^a^
Highest level of education before professional training									
Grade 7	41	6		40	6		52	8	
Form 4	41	7		41	6		54	7	
Form 6	39	12	0.353^b^	41.5	7	0.517^b^	55	8	0.376^b^
Time in current profession (in years)									
Less than 1 year	44	8		42	4		55	8	
1 to 3 years	40	7		41	7		54	7	
4 to 6 years	39	7		39	7		54	7	
7 to 12 years	41	8		40	7		55	8	
≧13 years	42	6	0.027^b^	41.5	7	0.036^b^	53	6	0.253^b^
Time in current health facility (in years)									
Less than 1 year	44.5	8		41.5	6		53	7	
1 to 3 years	41	7		41	7		54	7	
4 to 6 years	41	11		41	6		54	9	
7 to 12 years	40	8		40.5	7		54	6	
≧13 years	41	8	0.212^b^	40	8	0.999^b^	53	4	0.782^b^
Received training during the last 12 months									
No	42	7		42	6		53	7	
Yes	41	7	0.152^a^	40	7	0.071^a^	54	7	0.307^a^

^a^Mann Whitney U test

^b^Kruskal-Wallis test

In terms of Individual Aspects, workers on the Tanzania Government Health Scale (TGHS) (i.e., all health workers who participated in this study except medical attendants; take-home salary range per month: 250,000 to 1,313,000 TZS, mean salary per month 627,453 TZS) had higher motivation scores than workers on the Tanzania Government Health Operational Scale (TGHOS) (i.e., an operational category that includes medical attendants; take-home salary range per month: 135,500 to 620,000 TZS, mean salary per month 330,324 TZS) salary scale, and the difference was significant. Similarly, the motivation scores for Management and Performance Aspects of workers on the TGHS were higher than those on the TGHOS although the differences were not significant.

The highest level of education showed reverse associations on Management and Individual Aspects. For Management Aspects, the more education the health worker had, the lower his/her motivation scores became. On the contrary, for Individual Aspects, the more education the health worker had, the more motivated they were, although the differences were not statistically significant. Similarly, while there was no statistical significance, workers who did not attend any kind of training during the 12 months prior to the survey had higher motivation scores in Management and Performance Aspects than those who participated in training more than once.

The Pearson correlation coefficient was 0.45 between Management and Performance Aspects and 0.43 between Performance and Individual Aspects. The lowest coefficient was 0.19 between Individual and Management Aspects (all coefficients were significant at the 0.01 level, data not shown).

Table 5 shows the result of multivariate regression analysis. Health workers with a job description had higher motivation scores in both Management and Performance Aspects than those without a job description (both significant at 0.001 level). Likewise, the motivation scores in Management Aspects of health workers who were single, widowed, or separated were higher than those who were married or living with a partner (β: -1.85, 95% CI: -3.23 to -0.47, p = 0.009). For bivariate analysis, those working less than one year and 13 years or more had higher motivation scores in both Management and Performance Aspects than those working 1–12 years. Similarly, our multivariate analysis showed that those working less than one year and seven years or more had higher motivation scores in Management Aspects only (β: 1.41, 95% CI: 0.05 to 2.79, p = 0.043). In terms of motivation scores in Individual Aspects, workers who were on the TGHS salary scale had higher motivation scores than workers on the TGHOS salary scale (β: 1.52, 95% CI: 0.15 to 2.89, p = 0.029).

**Table 5:** Linear regression model for the predictors of health worker motivation score.

	Management Aspects				Performance Aspects				Individual Aspects			
	β	Standard Error	95% CI	P value	β	Standard Error	95% CI	P value	β	Standard Error	95% CI	P value
Marital status (married, cohabitation vs single, widowed, separated)	-1.85	0.70	-3.23 to -0.47	0.009								
Years worked in current profession (less than 1 year vs between 1 and 6 years)	2.93	1.43	0.11 to 5.74	0.042								
Years worked in current profession (between 1 and 6 years vs more than 7 years)	1.41	0.70	0.05 to 2.79	0.043								
Having a job description (with job description vs without job description)	2.85	0.70	1.47 to 4.22	<0.001	3.17	0.65	1.89 to 4.46	<0.001				
Salary scale (TGHS vs TGOHS)									1.52	0.69	0.15 to 2.89	0.029

Factor analysis using the principal axis method with a varimax rotation yielded the results presented in Appendix 3. After a number of iterations, 26 out of 45 items had a factor loading value greater than 0.4. As a result, eight factors were extracted as follows: Factor 1 (job satisfaction), Factor 2 (personal performance), Factor 3 (conscientiousness), Factor 4 (pride and commitment), Factor 5 (self-efficacy), Factor 6 (work organization), Factor 7 (aspiration), and Factor 8 (competency). These factors explained 36.8% of variance. The result of factor analysis showed that Management Aspects corresponded to Factor 5 (self-efficacy), Factor 6 (work organization), and Factor 8 (competency). Performance Aspects corresponded to Factor 2 (personal performance), Factor 4 (pride and commitment), and Factor 7 (aspiration). Individual Aspects corresponded to Factor 1 (job satisfaction) and Factor 3 (conscientiousness), revealing the validity of the tools based on Prytherch et al. (2012) [40].

## Discussion

Our sample might have been skewed toward female health workers compared to the sex-disaggregated current staff availability data at health centers and dispensaries. According to the Comprehensive Council Health Plan 2013–2014 of four study districts, the percentage of current male and female health workers of the four study districts combined was 46% and 54% respectively. Despite these limitations, this study offers details on three aspects of motivation of health workers who are posted in rural areas of mainland Tanzania.

The results of our regression analysis demonstrated that job description was the key variable for health worker motivation in both Management and Performance Aspects. A job description is defined as “a document, on file, that states the job title, describes the responsibilities of the position, the direct supervisory relationships with other staff, and the skills and qualifications required for the position [46].” A number of studies have revealed a positive relationship between well-defined roles and responsibilities of health workers and their performance [1,11,22,47,48], and that health workers with a job description have greater confidence in their roles and responsibilities [27]. In our study, 39% of the participants claimed that they did not have a job description or that they did not know whether they had one. According to CHMT members, reasons for not having a job description include the health worker not being given a written job description from the district medical officer, or the health worker receiving a job description but not recognizing it as such [39]. In some instances, job descriptions are given verbally rather than in writing [49,50].

Our analysis found that motivation scores in Individual Aspects were associated with salary scale. The difference in the mean salary between two salary scales (TGHS and TGHOS) was almost 300,000 TZS per month. Workers on the higher TGHS salary scale (i.e., all professions except medical attendants) had higher motivation scores in Individual Aspects than workers on the lower TGHOS scale (i.e., medical attendants). Medical attendants are on the lower end of the government salary scale. Their main duty is cleaning and other manual jobs [51]; yet, it is not uncommon to see them performing clinical tasks to meet the demands of patients, particularly in settings where a limited number of health workers are deployed [52]. A study conducted in Tanzania involving 566 health workers from 54 health facilities revealed that task shifting was fairly common. Workers whose tasks were delegated to lower categories were mainly medical officers and assistant medical officers. Then, their tasks were shifted to nurses and going down the ladder eventually to medical attendants [50]. No matter how many tasks medical attendants perform in addition to the tasks specified in their job description, their salaries remain the same and are the lowest among all health workers [10,53]. Furthermore, while health workers are to be promoted every three years under existing government regulations, medical attendants can be promoted only three times throughout their career, which means that the medical attendants who succeed in being promoted every three years will reach the maximum salary level within 10 years of their employment. Other studies show that this negatively affects working morale among medical attendants compared to other categories of health workers [10,53,54].

In our analysis, those who had been working less than one year and those who had worked for seven years or more had higher scores in the Management Aspects than those whose durations of work experience were between one and six years. A study in Papua New Guinea, which measured job satisfaction among rural nurses using a self-administered questionnaire, reported that the longer the duration in a profession, the higher the levels of job satisfaction among rural primary care nurses [55]. Results from other studies showed that time at a post, rather than time in the profession, predicted motivation [31,40,56]. In our study, the number of years the participant had served in the current profession, rather than in the current post, was associated with motivation. Furthermore, there was no linear relationship between motivation and number of years in the current profession. Health workers during their first year of professional service had higher motivation scores in Management Aspects. By the end of their first year, their level of motivation was seen to diminish, but by the time they reached the seventh year of their career, their level of motivation in Management Aspects was observed to increase. The higher motivation scores among the first-year health professionals can be explained by the fact that they may accept the working environment and conditions because it is their first post and they do not have any experience working at other facilities for comparison.

Our results did not show any significant difference in the motivation scores between those who had participated in workshop(s) and those who did not, contrary to findings from other studies [9,11,31,32,57]. According to previous studies, medical attendants were demotivated because they were often the ones who remained at the facility to cover for the more skilled health workers when they left to participate in training activities [10,53]. In our study, 168 (63.5%) of our participants, which included 58 (64.4%) medical attendants, participated at least one workshop during the 12 months preceding the time of the survey. While this percentage of medical attendants was lower than that for public health nurses, laboratory assistants, nurse midwives, and assistant nursing officers, it was higher than that of clinical officers and assistant medical officers. That is, medical attendants were not excluded from participating in various in-service training opportunities.

The major limitation of this study was that we did not verify the actual working conditions/environment that may have affected health worker motivation (including patient load, availability of essential medicines and medical equipment, frequency of supervision by Council Health Management Teams, etc.). Obtaining these data would have provided more insightful interpretation and analysis.

## Conclusions

This study measured three aspects of motivation among health workers in rural posts. The results showed that motivation was associated with marital status, having a job description, and number of years in the current profession for Management Aspects, having a job description for Performance Aspects, and salary scale for Individual Aspects. This study confirmed the importance of a written job description regardless of whether it reflects the actual tasks that a health worker is required to perform. Having a clear job description motivates health workers, and it can also be used as a human resource management tool by supervisors. The ongoing initiatives for the Human Resources for Health “Big Results Now” project in the Tanzanian health sector aim to achieve a 100% balanced distribution of skilled health workers at the primary level by 2017/18 [58]. One of the measures to achieve this goal is to “enhance the Open Performance Review and Appraisal System (OPRAS) and link it with recognition and reward [58].” With reinforcement of OPRAS, job descriptions will be recognized and routinely used as a performance management tool.

As Tanzania moves toward equitable distribution of human resources for health within and across regions, the motivation of health workers may improve in the next few years.

## Supporting information

S1 Appendix Formula used in sample size calculation. (DOCX)

S2 Appendix Questionnaire: Part II management aspects, performance aspects, and individual aspects of motivation. (DOCX)

S3 Appendix Summary of factor analysis. (DOCX)
