# Correction: Measuring three aspects of motivation among health workers at primary level health facilities in rural Tanzania

**DOI:** 10.1371/journal.pone.0184599

**Published:** 2017-09-06

**Authors:** Miho Sato, Deogratias Maufi, Upendo John Mwingira, Melkizedeck T. Leshabari, Mayumi Ohnishi, Sumihisa Honda

The fourth author’s name is spelled incorrectly. The correct name is: Melkizedeck T. Leshabari.
