# Supplementary material for: Measuring three aspects of motivation among health workers at primary level health facilities in rural Tanzania
Source: PLoS One. 2017 May 5;12(5):e0176973. doi: 10.1371/journal.pone.0176973 (PMC5419572; doi:10.1371/journal.pone.0176973)
Supplement: S1 Appendix — (DOCX) [file pone.0176973.s001.docx]

In order to have statistically significant correlation coefficient between teamwork scores and motivation scores, we expect r =0.3, alpha (two-sided) = 0.05, beta (one-sided) =0.2.

$$N=\left( \frac{Z_{\alpha}+Z_{\beta}}{C} \right)^{2}+3$$

$$Z_{\alpha}=1.96, Z_{\beta} =0.84$$

$$∁=0.5\times ln\frac{1+r}{1-r}$$

$$∁=0.31$$

$$N=84.5$$

Based on sample size calculation above, we will need at least 85 samples from each district. Therefore, the total sample size will be 170.
