# Supplementary material for: Measuring three aspects of motivation among health workers at primary level health facilities in rural Tanzania
Source: PLoS One. 2017 May 5;12(5):e0176973. doi: 10.1371/journal.pone.0176973 (PMC5419572; doi:10.1371/journal.pone.0176973)
Supplement: S2 Appendix — (DOCX) [file pone.0176973.s002.docx]

| **I. Management Aspects** |  |  |  |
| --- | --- | --- | --- |
| Construct | no. | Statement | Source/Remarks |
| Work organization | 1 | This facility provides everything I need to perform well at work | Adapted from [26] |
|  | 2 | There are enough health providers to do the work in this facility | [24] |
|  | 3 | Too often the referral system does not work efficiently |  |
|  | 4 | Maintenance of broken equipment at this facility is prompt and reliable |  |
|  | *5* | *The supply of medicine in this facility is not good* | New addition; Developmed by authors |
|  | *6* | *The supply of medicines has improved since last year* | New addition; Developmed by authors |
| Competence strengthening | 7 | My job duties and responsibilities are clear and specific | Adapted from [26] |
|  | 8 | Relevant guidelines are easy to access at this facility |  |
|  | 9 | I often feel left alone when I have to make difficult decisions about a patient’s care |  |
|  | 10 | I regularly have access to relevant trainings to keep my skills up to date |  |
| Role of performance | 11 | My performance is appraised regularly |  |
|  | 12 | Promotions do not depend on how well or badly one works on the job | Adapted from [26] |
| Self-efficacy | 13 | It is difficult for me to speak openly to my superiors about how things are really going at work | Adapted from [26] |
|  | 14 | Suggestions made by health workers on how to improve the facility are generally ignored | [24],[26] |
| Provider feels valued/exploited | 15 | The facility management shows very little concern for me | [26] |
|  | 16 | Our rights as health workers are generally not respected |  |
| **II. Performance Aspects** |  |  |  |
| Competence strengthening | 17 | I do not get feedback from my supervisors so it is hard to improve my performance | Adapted from [26] |
|  | 18 | The feedback I get from my co-workers helps me to improve my work | Adapted from [26] |
|  | *19* | *The feedback I get from CHMT helps me to improve my work* | New addition; Based on [52] |
| Role of performance | 20 | Good performance is recognized by our superiors | Adapted from 26 |
|  | 21 | This facility has a fair system for rewarding staff |  |
|  | 22 | Some of the team members work well, yet others do not and so this facility doesn’t perform well overall |  |
|  | 23 | We do not know how our facility is performing compared to others in the district |  |
|  | 24 | Our facility has clear goals that we are working towards |  |
|  | 25 | I am keen to use any new tools to improve my performance |  |
|  | 26 | This facility has a good reputation in the community | Adapted from [26] |
| Meaningfulness | 27 | I understand how my work contributes to the facility’s overall goals |  |
| Attitudes to patients | 28 | It makes me feel appreciated when patients are grateful |  |
| Pride/shame | 29 | I am proud to be working for this health facility | [24] |
|  |  | *I am proud to tell others that I work in maternal and neonatal health care* | removed |
| **III. Individual Aspects** |  |  |  |
| Self-efficacy | 30 | I usually cope well with changes at work | Adapted from [24] |
| Commitment | 31 | I intend to leave this facility as soon as I can find another position | Adapted from [24] |
|  | 32 | I would recommend to my children that they choose *my* profession | Adapted from [24] |
|  | 33 | I am willing to put in a great deal of effort to make this facility successful | Adapted from [26] |
| General & intrinsic motivation | 34 | These days I feel motivated to work as hard as I can | Adapted from [24] |
|  | 35 | My profession helps me to achieve my goals in life |  |
| Job satisfaction | 36 | Overall, I am very satisfied with my work *in this facility* |  |
|  | 37 | I am very satisfied to have a position where one works closely with the community |  |
|  | 38 | This job gives me a feeling of achievement and accomplishment | [26] |
| Timeliness and attendance | 39 | I am punctual about coming to work | [26] |
|  | 40 | I work hard to make sure that no patient has to wait a long time before being seen |  |
| Conscientiousness | 41 | I am careful not to make errors at work | [26] |
|  | 43 | When I am not sure how to treat a patient’s condition I look for information or ask for advice |  |
| Competency | *42* | *I have received enough training to perform the task that I am expected to do* | New Addition; Adapted from [45] |
| Cooperativeness | 44 | I try to get on well with the other health staff because it makes the work run more smoothly | Adapted from [26] |
|  | 45 | I get along well with my superiors at work | [26] |

All statements except new additions were from [40]. The sources indicated in the right column are the litereature cited in [40]. Those written in italics indicate new additions or adaptations for this study.
