# Supplementary material for: Measuring three aspects of motivation among health workers at primary level health facilities in rural Tanzania
Source: PLoS One. 2017 May 5;12(5):e0176973. doi: 10.1371/journal.pone.0176973 (PMC5419572; doi:10.1371/journal.pone.0176973)
Supplement: S3 Appendix — (DOCX) [file pone.0176973.s003.docx]

| **Factor** |  | **Factor Loadings** | | | | | | | |
| --- | --- | --- | --- | --- | --- | --- | --- | --- | --- |
|  |  | 1 | 2 | 3 | 4 | 5 | 6 | 7 | 8 |
| 1. Job satisfaction | 2_38_This job gives me a feeling of achievement and accomplishment. | .587 |  |  |  |  |  |  |  |
|  | 2_36_Overall, I am very satisfied with my work in this facility | .577 |  |  |  |  |  |  |  |
|  | 2_42_I have enough training to provide care to patients | .534 |  |  |  |  |  |  |  |
|  | 2_40_I work hard to make sure that no patient has to wait a long time before being seen. | .533 |  |  |  |  |  |  |  |
|  | 2_35_My profession helps me achieve my goals in life | .526 |  |  |  |  |  |  |  |
|  | 2_34_These days I have the morale to work as hard as I can. | .478 |  |  |  |  |  |  |  |
|  | 2_37_I am very satisfied to have a position where one works closely with the community. | .435 |  |  |  |  |  |  |  |
| 2. Personal performance | 2_23R_We do not know how our facility is performing compared to others in the district |  | .651 |  |  |  |  |  |  |
|  | 2_22R_Some of the team members work well, yet others do not and so this facility doesn't perform well overall |  | .592 |  |  |  |  |  |  |
| 3. Conscientiousness | 2_44_I try to get on well with the other health staff because it makes the work run more smoothly |  |  | .613 |  |  |  |  |  |
|  | 2_43_When I am not sure how to treat a patient's condition I look for information or ask for advice. |  |  | .474 |  |  |  |  |  |
|  | 2_28_It makes me feel appreciated when patients are grateful |  |  | .463 |  |  |  |  |  |
|  | 2_41_I am careful not to make errors at work. |  |  | .443 |  |  |  |  |  |
| 4. Pride and commitment | 2_29_I am proud to be working for this health facility |  |  |  | .815 |  |  |  |  |
|  | 2_26_This facility has a good reputation in the community |  |  |  | .516 |  |  |  |  |
|  | 2_31R_I intend to leave this facility as soon as I can find another position |  |  |  | .410 |  |  |  |  |
| 5. Self efficacy | 2_13R_It is difficult for me to speak openly to my superiors about how things are really going at work |  |  |  |  | .538 |  |  |  |
|  | 2_14R_Suggestions made by health workers on how to improve the facility are generally ignored |  |  |  |  | .528 |  |  |  |
|  | 2_16R_Our rights as health workers are generally not respected |  |  |  |  | .477 |  |  |  |
| 6. Work organization | 2_6_Availability of drugs and equipment has improved in the past year |  |  |  |  |  | .535 |  |  |
|  | 2_1_This facility provides everything I need to perform well at work. |  |  |  |  |  | .491 |  |  |
| 7. Aspiration | 2_25_I am keen to use any new tools to improve my performance |  |  |  |  |  |  | .552 |  |
|  | 2_24_Our facility has clear goals that we are working towards |  |  |  |  |  |  | .505 |  |
| 8. Competency | 2_9R_I often feel left alone when I have to make difficult decisions about a patient's care. |  |  |  |  |  |  |  | .474 |
|  | % of variance | 14.0 | 7.1 | 3.5 | 3.3 | 2.7 | 2.3 | 2.0 | 1.8 |

Extraction Method: Principal Axis Factoring. Rotation Method: Varimax with Kaiser Normalization.
